# Evaluating Gait Quality in People with Hip Osteoarthritis During Habitual and Fast Walking Using a Trunk Inertial Measurement Unit in Clinical Settings

**DOI:** 10.3390/s26030820

**Published:** 2026-01-26

**Authors:** Jiahui Wang, Abner Sergooris, Kristoff Corten, Annick A. A. Timmermans, Benedicte Vanwanseele

**Affiliations:** 1 Human Movement Biomechanics Research Group, Department of Movement Sciences, KU Leuven, 3001 Leuven, Belgium; benedicte.vanwanseele@kuleuven.be; 2 Faculty of Rehabilitation Sciences, Hasselt University, 3500 Diepenbeek, Belgium; abner.sergooris@uhasselt.be (A.S.); kristoff.corten@uhasselt.be (K.C.); annick.timmermans@uhasselt.be (A.A.A.T.)

**Keywords:** hip osteoarthritis, gait quality, nonlinear analysis, a single trunk IMU, wearable sensor, gait symmetry, gait stability, gait smoothness, gait regularity, gait complexity

## Abstract

**Highlights:**

**What are the main findings?**

- Hip osteoarthritis reduces gait symmetry and stability and shows vertical gait impairments during habitual walking
- At fast walk, hip osteoarthritis reduces step symmetry but not stability

**What are the implications of the main findings?**

- A single trunk sensor effectively captures hip osteoarthritis gait quality in clinics
- IMU-derived gait quality parameters provide additional biomechanical detail beyond speed-based clinical tests

**Abstract:**

Hip osteoarthritis (OA) affects the entire joint and significantly alters gait. Assessing gait through a single trunk inertial measurement unit (IMU) in clinical settings offers a more practical alternative to complex laboratory settings, allowing for the capture of natural gait movements with valuable biomechanical insights. We evaluated (1) whether gait quality differs between individuals with hip OA and healthy controls during habitual and fast walking, (2) whether gait changes from habitual to fast walking differ between groups. Forty individuals with hip OA and 40 age-matched healthy controls underwent 25-m habitual walk and 40-m fast walk. Six gait quality parameters—step symmetry, stride symmetry, stability, smoothness, regularity, and complexity—were analyzed from the IMU signals. During habitual walking, individuals with hip OA exhibited reduced symmetry and stability and several vertical impairments. During fast walking, individuals with hip OA continued to show reduced step symmetry and a more constrained gait in the mediolateral direction. Additionally, people with hip OA also showed limited adjustments when transitioning from habitual to fast walking, in contrast to the significant adjustments observed in healthy controls. These findings indicate that gait in individuals with hip OA is impaired during habitual and fast walking, with limited adaptations across the transition between the two conditions.

## 1. Introduction

Hip osteoarthritis (OA) is an increasingly common and debilitating condition, representing a growing threat to global health [1]. From 1990 to 2019, the global incidence more than doubled from 0.74 million to 1.58 million cases, marking an increase of 115.40% [2]. It is estimated that every one in four people will likely develop symptomatic hip OA by the age of 85 [3]. Hip OA is a whole joint disorder, characterized by degenerative joint cartilage and alterations in subchondral bone, synovium, joint capsule, ligaments, and periarticular muscles. This whole joint involvement can lead to pain, stiffness, and functional limitations, making everyday activities challenging [1]. The notable effects of hip OA on mobility are primarily observed through significant changes in gait [4]. This emphasizes the need to explore how hip OA specifically impacts gait, a critical element of daily mobility.

Previous research indicated that individuals with hip OA typically have a shorter step length and walk 26% slower than healthy individuals [4]. Due to their limited hip range of motion, individuals with hip OA tend to exhibit a faster cadence and reduced step length to maintain pace [5]. Additionally, these persons adopt strategies such as a larger step width and a shorter single support phase to reduce pain and loading on the affected joint [4,6]. While these adaptations can reduce discomfort in the short term, they also lead to increased gait asymmetry over time, affecting both the affected and unaffected leg [6]. These modifications in gait parameters illustrate the considerable challenges faced by individuals with hip OA in maintaining normal gait locomotion.

Current methods for assessing gait in persons with hip OA predominantly rely on lab-based equipment, which presents significant limitations for use in clinical settings due to its complex setup and the need for specialized environments [7]. In contrast, clinical settings often utilize performance-based assessments such as the 40-m Fast Paced Walk Test (40m FPWT) as a more practical and highly reliable alternative for assessing walking function in patients with hip OA [8]. However, these tests lack detailed biomechanical insights that are critical for a more comprehensive analysis. Additionally, they may not accurately reflect a person’s natural movement behavior.

In the field of gait analysis, the use of inertial measurement units (IMUs) marks a pivotal advancement [9]. IMUs offer a portable solution that captures detailed biomechanical data while preserving the natural dynamics of walking movements. These systems, which integrate accelerometers, gyroscopes, and magnetometers, have proven particularly valuable in clinical settings where traditional lab-based equipment is impractical [10]. A notable application is the use of a single trunk IMU placed at the lower trunk level, which offers an unobtrusive way to assess several characteristics of gait biomechanics [10]. Single-trunk IMU gait analysis has been widely used in various populations, including elderly subjects, children, and individuals with neurological disorders such as Parkinson’s disease and stroke, as well as musculoskeletal diseases like orthopedic conditions and lower limb amputations [11,12,13,14,15]. The use of IMUs in clinical settings has increased recently, yet their application specifically for hip OA research remains limited.

Combining a single trunk IMU system with advanced signal processing, particularly nonlinear analysis, enables detailed characterization of gait quality beyond traditional measures [16], which is particularly relevant in individuals with hip OA. Symmetry metrics have been successfully used to detect differences between hip OA and healthy controls [12] and are also sensitive enough to identify subclinical gait alterations in pre-manifest Huntington’s disease carriers [17]. Gait instability, particularly in the mediolateral (ML) direction, has been reported in hip and knee OA [12], in Anterior Cruciate Ligament (ACL)-deficient, and ACL-reconstructed individuals [18,19]. Smoothness has shown sensitivity to aging-related gait changes and to early alterations in populations with central nervous system dysfunctions, such as multiple sclerosis [20]. Although not yet widely applied in hip OA, it may capture subtle disruptions in movement coordination due to pain-related compensations. Regularity is associated with impaired motor control and increased fall risk in populations with ACL reconstruction [19,21,22]. Lower complexity has been linked to higher fall risk in older adults [23,24]. Despite the clinical potential of these metrics, most existing studies have focused on a limited subset, typically in controlled environments and at a single walking speed. This study addresses these gaps by applying a full multidimensional gait analysis across multiple walking conditions to better understand functional deficits in OA and identify sensitive markers that could enhance current clinical evaluations.

Therefore, this study aimed to investigate (1) whether gait quality differs between persons with hip OA and healthy controls during habitual walking and fast walking, and (2) whether transitioning from habitual walking to fast walking induces different changes in gait quality between people with hip OA and healthy controls.

## 2. Materials and Methods

### 2.1. Study Design and Study Sample

This study included 40 persons with hip OA awaiting total hip arthroplasty surgery, who were included in a larger prospective longitudinal cohort study conducted at Hasselt University [25]. Ethical approval for this cohort was obtained from the medical ethics committee of Hospital East-Limburg and Hasselt University (B3712021000002). All participants were included based on a confirmed clinical or radiographic diagnosis of hip OA. Exclusion criteria included rheumatic diseases other than OA, avascular necrosis or other conditions explaining symptoms, significant neurological disorders (e.g., Parkinson’s disease, dementia), revision THA, history of pathological fractures, or planned surgeries in the one-year follow-up period. All assessments for the experimental group took place at Hospital East-Limburg in Genk, Belgium. Separate ethical approval was obtained for the healthy control group from the Ethics Committee Research (EC Research) UZ/KU Leuven (s68645). The control group was comprised of 40 age-matched healthy individuals who met the following criteria: aged 50 and above, Body Mass Index (BMI) under 30, the ability to walk 10 m unaided, and the capability to ascend and descend stairs. Exclusion criteria group were pain in the lower limbs, lower leg injuries, severe health conditions like heart failure or uncontrolled hypertension, recent major lower extremity surgeries, severe mobility impairments, or significant cognitive impairments. All exclusion criteria were assessed in person during a pre-measurement screening conducted prior to data collection and before obtaining written informed consent. Each exclusion criterion was systematically reviewed through a structured interview with participants. Cognitive status was assessed by confirming the absence of a self-reported diagnosis of cognitive or neurological impairment and by verifying the participant’s ability to understand study instructions and provide informed consent. Participants who did not meet the eligibility criteria were excluded. The control group performed all assessments in an indoor sporting hall (Building De Nayer, Leuven, Belgium). They completed the entire assessment twice, one week apart. For the main group-level analyses, only the first session was included for both groups. The second session for healthy participants was analyzed separately for reproducibility assessment and was not incorporated into the primary analyses.

### 2.2. Data Acquisition

**Figure 1:**
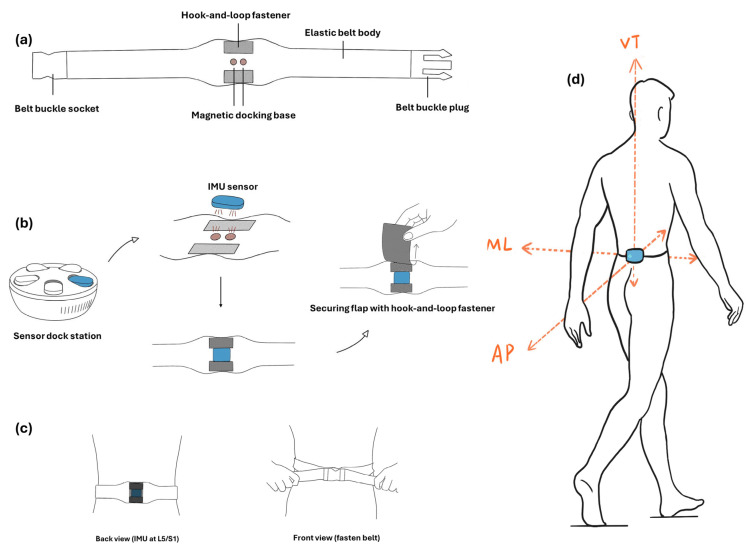
This figure illustrates participants equipped with a single trunk IMU (Byteflies, Antwerp, Belgium) positioned at the L5/S1 level. It captures acceleration and angular velocity across the three principal directions—vertical (VT), mediolateral (ML), and anteroposterior (AP). (**a**) Customized elastic belt with embedded magnets and hook-and-loop fasteners. (**b**) IMU magnetically attached and secured with a fabric overlay flap. (**c**) Ergonomic, adjustable belt fitted around the waist for stable positioning. (**d**) Final placement of the IMU at L5/S1.

At the start of the assessments, a single trunk IMU (Byteflies, Antwerp, Belgium; 200Hz, 16-bit, ACC: ±8 g; GYR: ±1000 degrees/s, 6.3 g) was attached to the lower back of each participant and recordings continued throughout the entire measurement. The IMU was secured using a customized waist belt designed for this study (Figure 1a). Embedded magnets precisely matched the sensor’s shape, ensuring a firm magnetic attachment that prevented rotation and positional changes. An additional fabric overlay further secured the sensor, eliminating the risk of accidental detachment (Figure 1b). The belt was tailored to follow the waist contour, and the belt length could be adjusted to accommodate different waist sizes, ensuring consistent positioning, minimal belt tension variation, and reducing potential movement artifacts (Figure 1c). Finally, the IMU was positioned at the L5/S1 level (Figure 1d). For the OA group, gait data were collected during a dedicated test session carried out by physiotherapists. Participants first performed the 40 m FPWT, in which they walked back and forth at maximal speed over a 10-m stretch marked by cones, completing four laps for a total of 40 m [8]. They then walked directly without additional instructions to a stair climbing task. On the way between test stations, they traversed a fixed 25-m straight indoor walkway. During this segment, habitual walking was captured. For the healthy control group, participants performed the same 40 m FPWT and also traversed a 25-m straight indoor walkway between test stations while wearing the IMU throughout the session, allowing extraction of habitual walking directly comparable to the OA group. Both cohorts completed the same assessment protocol in the same fixed order.

### 2.3. Data Processing

First, the sensor axis system was aligned to the global coordinate system, applying the triangulation method proposed by Moe-Nilssen et al. for tilt corrections [26]. Subsequently, segments of the fast and habitual walking signals were extracted, excluding parts of the data where participants turned, creating a continuous and stable time series from steady walking segments.

Step events were derived from the anteroposterior (AP) trunk acceleration signal using an automatic peak-detection approach based on the methodology of Zijlstra and Hof [27]. Characteristic peaks in the AP acceleration waveform were associated with successive step events, with each peak corresponding to one step. A stride was defined as two consecutive steps. In rare cases of gait asymmetry, two successive AP acceleration peaks occurred in close temporal proximity, causing the automatic peak-detection algorithm to identify only a single peak and thus missing one step. In these cases, manual inspection of the AP acceleration signal was performed to identify and add the missing step based on the presence and timing of the closely spaced AP peaks.

Afterward, the left and right steps were defined by the slope of the angular velocity around the vertical (VT) axis (yaw angular velocity). During walking, the sign of the yaw angular velocity was used to classify steps as left or right. No manual correction of step laterality was required.

All trials were visually checked to ensure accuracy. All visual inspections and manual corrections were performed by a single trained assessor following predefined criteria. In rare cases where pauses or interruptions occurred, these segments were manually excluded. The least number of strides observed among participants was 18, so the signal length of all participants was truncated to this number (±3600 data points).

### 2.4. Gait Quality Parameters

Gait quality was defined by six parameters: step symmetry, stride symmetry, stability, regularity, complexity, and smoothness.

Step and stride symmetry were assessed using autocorrelation to evaluate the similarity between the original signal and its one- and two-step delayed versions [17]. For step symmetry, the analysis considered the absolute value of the first dominant peak. Similarly, the second dominant peak represents stride symmetry. In both cases, the autocorrelation coefficient closer to 1 indicates higher symmetry.

Stability was quantified using the maximum Lyapunov exponent, which represents “the average exponential rate of divergence or convergence of nearby trajectories in state space” [28]. We implemented the Rosenstein algorithm [29], extended to multiple neighbors following Mehdizadeh [30]. The entire gait signal was resampled to a length of (number of strides × 200) data points, based on the sensor’s sampling frequency (200 Hz) and approximately 200 samples per stride (≈1 s). This resampling was applied once to the whole signal to preserve stride structure while normalizing length across participants. State-space reconstruction used embedding dimensions of 5 and time delay of 10. For each reference point, the 7 nearest neighbors were identified, excluding points within ±0.5 stride periods to avoid temporal correlation. The Euclidean distances between trajectories were tracked over a 10-stride window. Higher Lyapunov exponent values reflect faster divergence and thus lower dynamic stability, suggesting greater instability and an increased risk of falling.

Regularity and complexity were utilized by two related entropy measures. Regularity was measured by Sample Entropy (SaEn), quantifying the unpredictability in a signal [24]. We used the embedding dimension of 2 and a tolerance set to 0.2 times the time-series standard deviation, with the time-series length N matched that used in stability analysis. SaEn values ranged from nearly zero (high regularity) to infinite (low regularity). Complexity was measured using Multiscale Entropy (MSE) by generating multiple new time series from the original [31]. This process involves dividing the original series into non-overlapping windows, averaging the data within each window, and calculating SaEn for both the average series and the original. MSE was evaluated across 6 scales with higher MSE indicating greater complexity.

Gait smoothness was quantified using the Spectral Arc-Length Metric (SPARC), a robust, validated measure for gait analyses [22]. Angular velocity data (yaw, pitch, roll) were Fourier transformed to a frequency spectrum, then normalized to the spectrum’s maximum value for across-sample comparability. We used a 10 Hz cut-off frequency and an amplitude threshold set to 0.05 to determine the significant frequency components contributing to movement smoothness. SPARC was calculated by measuring the normalized arc length of this spectrum up to 10 Hz. SPARC values are conventionally reported as negative, smaller absolute values indicate smoother gait.

For visualization in the spider plots, sample entropy and the maximum Lyapunov exponent were inverted so that higher values correspond to greater regularity and stability, while tables report the original, non-transformed values.

All data processing was performed using MATLAB R2024b (MathWorks, Natick, MA, USA).

### 2.5. Statistical Analysis

The Intraclass Correlation Coefficient (ICC (3,1)) was calculated to assess test–retest consistency in healthy controls. All model assumptions (normality and homoscedasticity of residuals) were first assessed using visual inspection of Q–Q plots and residuals versus fitted plots.

To compare gait quality during habitual and fast walking between individuals with OA and healthy controls, each gait outcome was analyzed separately using linear regression including Group (OA vs healthy) as the predictor. To account for potential confounding by sex, all models were additionally adjusted for gender. We also adjusted for differences in speed by using Group as a fixed factor and Speed as a covariate alongside Group and gender.

To examine whether gait adaptations from habitual to fast pace differed between groups, linear mixed-effects models were fitted with Group (OA vs healthy), Condition (fast pace vs habitual pace), their interaction as fixed effects, and Participant as a random intercept to account for repeated measures. Walking speed was included as a covariate in the speed-adjusted analysis and gender was adjusted in all models.

For all analyses, *p*-values were adjusted for multiple comparisons using the Benjamini–Hochberg false discovery rate procedure. Statistical significance was set at *p* < 0.05. All analyses were conducted in R (version 4.4.1) using RStudio (version 2023.6.0.421, RStudio, Inc.).

## 3. Results

All gait-quality parameters demonstrated good to excellent test–retest reliability (Supplementary Table S1).

Subject characteristics are shown in Table 1. BMI was significantly higher in persons with hip OA and in their age-matched healthy controls. During both habitual and fast walking, persons with hip OA walked significantly slower. For both groups, walking speed at fast pace was significantly higher than at habitual pace.

**Figure 2:**
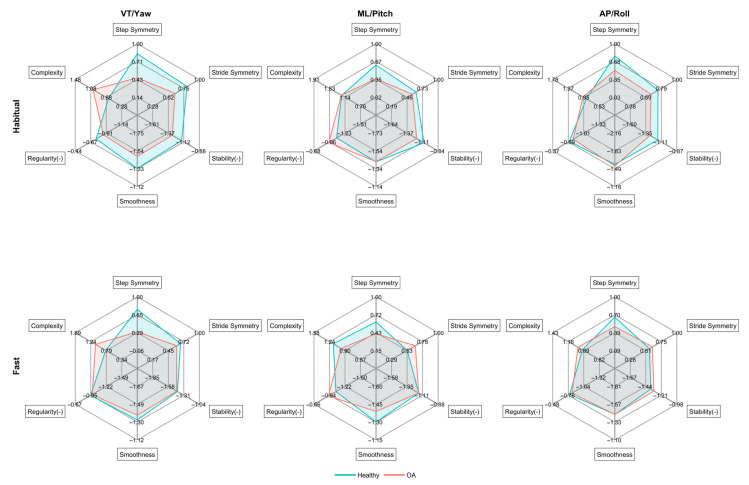
This figure displays spider plots to illustrate group differences in gait parameters, including step/stride symmetry, stability, smoothness, regularity, and complexity across the three principal directions—vertical (VT), mediolateral (ML), and anteroposterior (AP) as well as the corresponding rotational axes (yaw, pitch, and roll). Gait parameters including symmetry, stability, regularity, and complexity were calculated from acceleration signals and presented across the three translational directions: VT, ML, and AP, whereas gait smoothness was calculated from angular velocity signals and presented for the corresponding rotational axes: yaw, pitch, and roll. In these plots, persons with hip osteoarthritis (OA) are shown in red and healthy controls in blue. Each axis of the plot corresponds to a different gait parameter. The plots are designed such that points closer to the edge indicate increased levels of the respective parameter. Specifically, proximity to the edge denotes more symmetry, more stability, more smoothness, and more regularity in the gait patterns. The closer proximity to the edge uniformly represents a greater presence of that gait characteristic. For stability and regularity, negative values were utilized in the analysis; thus, more negative values indicated greater stability and regularity. This method was also applied to the step symmetry in the ML direction, where the absolute value was used to represent this measure.

Several gait quality parameters were different between persons with hip OA and age-matched healthy controls during habitual and fast walking (Figure 2). During habitual walking, persons with hip OA exhibited reduced step and stride symmetry in all three directions, indicating notable gait asymmetry (Table 2). Gait stability was also significantly reduced in persons with hip OA compared to healthy individuals. Moreover, persons with hip OA walked with lower regularity and higher complexity in the VT direction, along with higher regularity in the ML direction. In addition, persons with hip OA walked with less smoothness in yaw rotational motion. After adjusting for walking speed, most group differences were no longer significant. Only step symmetry in the ML direction remained significantly lower in persons with hip OA during habitual walking, suggesting this parameter reflects gait impairment beyond the influence of speed.

During fast walking, persons with hip OA showed lower step symmetry in all three directions. There were no significant differences in gait stability between persons with hip OA and healthy controls at fast walking. Persons with hip OA showed greater stride symmetry, increased regularity, and decreased complexity compared to healthy controls, along with higher complexity in the VT direction. In addition, persons with hip OA walked with less smoothness in yaw rotational motion. After adjusting for walking speed, step symmetry remained significantly lower in the OA group for all three directions. All significant differences in the ML direction remained. In the VT direction, complexity differences remained, while additional significant differences emerged, including lower stride symmetry and reduced yaw smoothness in persons with hip OA.

**Figure 3:**
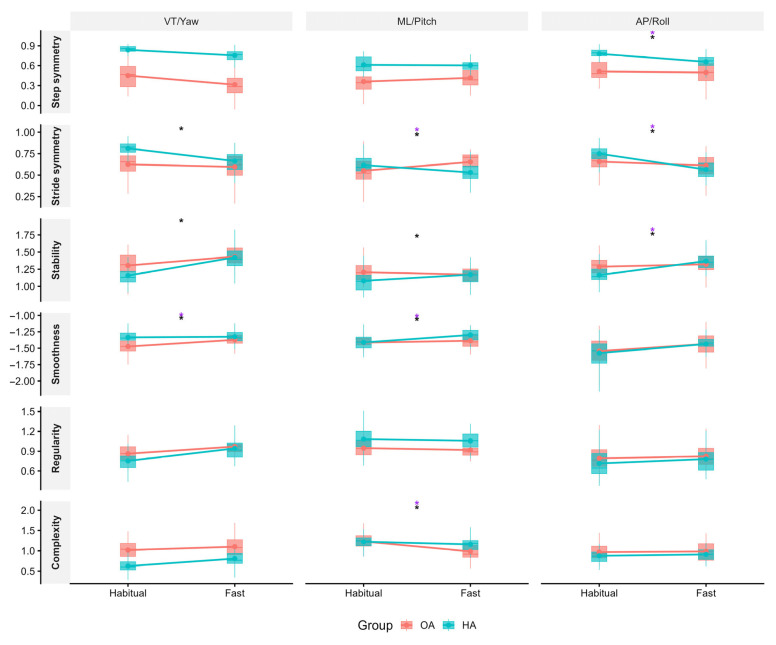
A comprehensive comparison of various gait parameters between the hip OA (Osteoarthritis) and control group during habitual and fast walking. The analysis includes six parameters assessed in three directions: vertical (VT)/yaw, mediolateral (ML)/pitch, and anteroposterior (AP)/roll. OA group data is represented in red, while healthy group data is represented in blue. A black ‘*’ indicates a significant interaction difference between the OA and healthy groups across the two speed conditions before speed correction, while a purple ‘*’ indicates a significant interaction difference after speed correction.

Significant interaction effects between Group (OA vs. healthy) and Condition (habitual vs. fast pace) were observed for several gait measures (Figure 3). For step symmetry, interaction effects were found in the AP direction. Stride symmetry and gait stability showed interaction effects across all three directions (VT, ML, and AP). Gait smoothness showed interaction effects in yaw and roll. For gait complexity, interaction effects were found in the ML direction. No interaction effects were observed for gait regularity in any direction. When walking speed was included as a covariate, significant interactions remained for AP step symmetry, ML and AP stride symmetry, yaw and roll smoothness, AP stability, and ML complexity.

## 4. Discussion

Based on this study’s findings, persons with hip OA walk significantly differently at their habitual pace compared to age-matched healthy controls, exhibiting less step and stride symmetry and lower gait stability across all three directions, with VT impairments present across all gait quality parameters. However, most of these differences were no longer significant after adjusting for walking speed. In contrast, during fast walking, hip OA showed lower step symmetry, but the stability of persons with hip OA did not differ from healthy individuals. Additionally, when transitioning to faster speeds, OA and control groups demonstrated different adjustment in gait quality. By examining six gait domains under two independent conditions, this study provides detailed biomechanical data on gait in hip OA, contributing to a more comprehensive understanding of disease-related gait alterations for clinical and research applications.

Persons with hip OA showed lower step and stride symmetry in all three directions compared to healthy people during habitual walking, which is consistent with earlier research findings [4,12]. All 40 participants in this study had unilateral end-stage hip OA, which typically results from degenerative changes that unevenly affect two sides of the hip joints, leading to asymmetries [32]. Rather than representing a mere deviation from normal gait, this asymmetry likely arises from pain, muscle weakness, and other sensorimotor deficits in the more affected limb, and may function as a compensatory adaptation to reduce mechanical load and mitigate pain on the affected side [33].

Persons with hip OA showed less stable gait across all directions during habitual walking. This aligns with existing literature, which consistently points to end-stage hip OA as a significant factor that markedly deteriorates overall stability and increases the risk of falls [34,35]. Laboratory studies further support this, showing impaired stability and elevated fall-risk indices even in mild to moderate disease [36]. In addition, individuals with unilateral hip OA performed worse on standardized functional and clinical stability measures such as the Berg Balance Scale and the Timed Up and Go test [37]. Consistently, nearly half of older adults with hip OA report at least one fall per year [38,39]. In contrast, some studies have reported no differences in gait stability between individuals with hip OA and healthy controls [12,40]. These studies were conducted under different conditions, such as walking 10 m in a laboratory at self-selected speed or with treadmill walking at controlled speeds and included participants with moderate rather than end-stage disease. Variations in experiment settings and disease stage may explain the different findings, as impairments in stability might be more apparent during habitual walking in individuals with end-stage hip OA.

Persons with hip OA walked with less regularity and higher complexity, together with reduced symmetry and stability in the VT. This aligns with previous studies demonstrating impairments in the VT direction in hip OA, reporting significantly lower step regularity [12,41]. Laboratory-based studies also indicated altered vertical loading patterns in hip OA, providing complementary evidence of gait differences in the vertical direction [42,43]. In addition, musculoskeletal modeling suggested disproportionate contributions of the gluteus maximus to the vertical center of mass acceleration in hip OA [44]. Our study complements this knowledge by showing that, during habitual walking, all domains of vertical gait quality are consistently impaired.

During fast walking, persons with OA continued to show marked gait asymmetry across all three directions. Interestingly, stability no longer differed between groups at fast walking. This suggests that OA persons were able to adapt by preserving stability under high demand, but they did so at the expense of other gait qualities mainly in the ML direction. Persons with OA showed greater regularity, higher stride symmetry, and lower complexity in the ML direction, indicating a more constrained and rigid gait movement. This is consistent with earlier research findings that persons with hip OA often display frontal-plane sideways compensations such as trunk lean and altered hip adduction moments as strategies to control stability [45]. These findings suggest that hip OA may alter ML gait movement in a way that may help preserve stability but results in more constrained movement during fast walking.

During both habitual and maximum speed walking, individuals with hip OA walked significantly slower than healthy controls. Although walking speed is a strong determinant of many gait parameters, it also reflects pain, functional limitation, and adaptive motor strategies that are intrinsic to the disease process. Therefore, both unadjusted as well as adjusted results are important as adjustment for walking speed may attenuate clinically meaningful between-group gait differences but can help to explore the extent to which observed impairments are velocity-dependent. After speed-adjustment, most group differences were no longer significant during the habitual walking, indicating that poorer gait quality in OA could be associated with persons slower habitual walking speed. Only ML step symmetry remained impaired, reflecting a gait deficit independent of speed. These results should be interpreted carefully as this does not mean that gait quality could simply be improved if individuals with OA walked faster as healthy persons. Although they were able to increase speed when explicitly instructed, transitioning to their fast walking revealed persistent and even greater asymmetry as well as some additional abnormalities in the ML and VT direction. These findings suggest that when challenged to walk closer to their physical limits, individuals with OA exhibit intrinsic gait control deficits that cannot be explained solely by slower speed. Moreover, in daily life, individuals with OA generally adopt slower self-selected speeds, likely due to pain, joint restriction, or fear of instability, and at these speeds they do exhibit less symmetrical and less stable gait. Overall, the results indicate that while some gait quality parameters in OA might be speed-dependent, there are impaired gait quality parameters representing disease-specific deficits that persist across walking speeds.

When comparing habitual with fast walking, differences between OA and healthy controls were found in the AP direction for step symmetry, stride symmetry, and stability, even correcting for speed. Post hoc contrasts showed that healthy adults exhibited significant decreases in AP step and stride symmetry and less stability when transitioning from habitual to fast walking, whereas the OA group did not change in any of these measures. These results are consistent with previous studies demonstrating comparable declines in dynamic stability and gait symmetry among healthy adults when speed increases [46,47]. In contrast, individuals with hip OA showed no significant change in AP symmetry or stability when transitioning to fast walking. This might be due to the fact that OA individuals’ stability and symmetry at habitual walking were already comparable to the reduced levels observed in healthy individuals during fast walking. In other words, OA individuals operate at a lower baseline of AP stability and symmetry at habitual speeds, and unlike healthy adults, they do not adapt further when challenged by speed demands. This pattern reflects a potential limitation in their adaptive capacity in the AP direction and may indicate underlying impairments in AP control. Supporting this interpretation, previous studies demonstrated that individuals with hip OA exhibit diminished hip-generated forward propulsion as well as reduced ankle push-off power and overall propulsive force compared to healthy adults [48,49].

Our study has several limitations that should be considered when interpreting the results. Notably, there was a significant difference in BMI between the healthy and OA groups. As increased BMI is associated with the development and progression of OA [50], recruiting BMI-matched healthy controls is challenging. Additionally, the healthy participants recruited for this study were particularly fit and free from pain to minimize the influence of pain on gait patterns, characteristics that may not be typical of the general population of similar age. This discrepancy in physical condition could lead to an overestimation of the differences in gait parameters between healthy individuals and those with OA, as the healthy group’s superior physical condition might not accurately represent an average older adult. Pain severity was not included as a covariate due to the limited sample size, and physical activity was not assessed and therefore could not be accounted for. These factors suggest that while our findings provide valuable insights, they should be generalized with caution to the broader population. Further studies are needed to confirm these findings across a more representative sample of middle-aged and elderly adults.

## 5. Conclusions

This study found that persons with hip OA walk differently from healthy controls across multiple gait quality parameters. During habitual walking, the OA group showed less symmetry and stability, with VT impairments present across all gait quality parameters. At fast walking, hip OA individuals showed decreased step symmetry and more constrained ML movement, while their stability was at the same level as healthy controls. When transitioning from habitual to fast walking, the healthy group experienced significant reductions in AP symmetry and stability, whereas the OA group showed no changes. This study provides biomechanical data that may support both clinical assessment and future research as the findings augment the interpretation of gait data beyond traditional speed-based tests such as the 40MFW, supporting the development of more personalized treatment strategies. As there is currently no evidence on how specific gait parameters can be modified through interventions in hip OA or related populations, further research is needed to expand on these results and address the distinct gait challenges faced by individuals with hip OA.

## Figures and Tables

**Table 1:** Descriptive demographic, clinical, radiographic, functional and walking speed characteristics of hip Osteoarthritis (OA) group and control group. Continuous variables are reported as mean (SD) and categorical variables as counts (*n*). *p*-values correspond to between-group comparisons using two-sampled *t*-test where applicable. Pain intensity reflects the average hip pain experienced over the previous week, assessed using a visual analogue scale. Radiographic severity is presented as Tönnis grade distribution (*n*). The stair climbing test was assessed in the OA group only.

	OA	Controls	*p* Value
Sex (*n* = female; *n* = male)	19; 21	27; 13	-
Age (years)	65 ± 8	65 ± 8	1
Body mass index (kg/m^−2^)	27.8 ± 6.5	24.5 ± 3.6	**0.01**
Habitual walking speed (m/s)	0.64 ± 0.08	1.38 ± 0.15	**0.00**
Fast walking speed (m/s)	1.62 ± 0.45	1.8 ± 0.17	**0.03**
Radiographic severity (Tönnis grade 1; 2; 3)	6; 9; 25	-	-
Affected side (*n* = left; *n* = right)	15; 25	-	-
Pain intensity (visual analogue scale, 0–10)	5.16 ± 1.98	-	-
Hip disability and Osteoarthritis Outcome Score	44.63 ± 21.57	-	-
40 m fast walking test (s)	28.55 ± 8.08	23.55 ± 2.37	**0.00**
30 s sit-to-stand test (repetitions)	11.11 ± 4.68	26.05 ± 6.81	**0.00**
Stair climbing test (s)	13.39 ± 7.07	-	-

**Significant *p* values (*p* < 0.05) are shown in bold.**

**Table 2:** Gait quality measures in VT/Yaw, ML/Pitch, and AP/Roll directions during habitual- and fast-walking in hip osteoarthritis (OA) and control groups, before and after speed correction.

Habitual Walking							
		OA	Control	Estimate *Before Speed Correction*	*p* Value *Before Speed Correction*	Estimate *After Speed Correction*	*p* Value *After Speed Correction*
Step symmetry	VT	0.451 (0.196)	0.839 (0.077)	−0.374	**<0.001**	−0.271	0.070
	ML	0.361 (0.146)	0.612 (0.117)	−0.232	**<0.001**	−0.260	**0.039**
	AP	0.513 (0.166)	0.780 (0.092)	−0.259	**<0.001**	−0.158	0.285
Stride symmetry	VT	0.625 (0.149)	0.812 (0.073)	−0.174	**<0.001**	−0.100	0.368
	ML	0.551 (0.161)	0.616 (0.104)	−0.045	**0.049**	−0.040	0.744
	AP	0.657 (0.119)	0.750 (0.087)	−0.080	**<0.01**	−0.094	0.354
Stability	VT	1.304 (0.180)	1.158 (0.143)	0.148	**<0.001**	−0.075	0.655
	ML	1.206 (0.149)	1.081 (0.166)	0.144	**<0.001**	0.174	0.285
	AP	1.287 (0.154)	1.165 (0.147)	0.124	**<0.01**	0.074	0.655
Smoothness	Yaw	−1.474 (0.123)	−1.334 (0.096)	−0.138	**<0.001**	−0.056	0.655
	Pitch	−1.415 (0.118)	−1.412 (0.114)	0.012	0.716	0.123	0.285
	Roll	−1.544 (0.206)	−1.573 (0.195)	0.019	0.720	0.198	0.331
Regularity	VT	0.863 (0.146)	0.753 (0.131)	0.103	**<0.01**	0.153	0.285
	ML	0.948 (0.126)	1.084 (0.191)	−0.145	**<0.001**	−0.196	0.285
	AP	0.793 (0.208)	0.715 (0.218)	0.073	0.168	−0.082	0.713
Complexity	VT	1.019 (0.235)	0.624 (0.158)	0.376	**<0.001**	0.219	0.285
	ML	1.231 (0.194)	1.224 (0.217)	−0.003	0.954	0.021	0.939
	AP	0.970 (0.187)	0.878 (0.220)	0.082	0.111	−0.007	0.963
Fast walking							
Step symmetry	VT	0.313 (0.202)	0.755 (0.082)	−0.438	**<0.001**	−0.461	**<0.001**
	ML	0.417 (0.145)	0.603 (0.094)	−0.176	**<0.001**	−0.184	**<0.001**
	AP	0.497 (0.174)	0.657 (0.101)	−0.155	**<0.001**	−0.206	**<0.001**
Stride symmetry	VT	0.595 (0.170)	0.664 (0.120)	−0.063	0.113	−0.078	**0.048**
	ML	0.653 (0.118)	0.531 (0.115)	0.126	**<0.001**	0.111	**<0.01**
	AP	0.612 (0.145)	0.565 (0.106)	0.052	0.113	0.042	0.220
Stability	VT	1.434 (0.135)	1.422 (0.184)	0.025	0.533	0.021	0.651
	ML	1.171 (0.108)	1.170 (0.145)	0.01	0.759	0.004	0.902
	AP	1.322 (0.145)	1.369 (0.125)	−0.039	0.287	−0.057	0.132
Smoothness	Yaw	−1.372 (0.108)	−1.325 (0.093)	−0.039	0.113	−0.049	**0.048**
	Pitch	−1.386 (0.123)	−1.298 (0.088)	−0.081	**<0.01**	−0.081	**0.01**
	Roll	−1.435 (0.182)	−1.436 (0.136)	0	0.996	0.015	0.744
Regularity	VT	0.971 (0.142)	0.943 (0.184)	0.031	0.488	0.060	0.157
	ML	0.919 (0.162)	1.057 (0.144)	−0.141	**<0.001**	−0.143	**<0.01**
	AP	0.823 (0.180)	0.781 (0.200)	0.045	0.375	0.100	0.052
Complexity	VT	1.101 (0.255)	0.808 (0.244)	0.305	**<0.001**	0.326	**<0.001**
	ML	0.985 (0.215)	1.159 (0.171)	−0.166	**<0.001**	−0.159	**<0.01**
	AP	0.986 (0.238)	0.912 (0.143)	0.081	0.113	0.094	0.09

VT: Vertical; ML: Medial–lateral; AP: Anteroposterior. This table reported the original, non-transformed values; only step symmetry (ML) is shown as absolute values for comparability. Significant *p* values (*p* < 0.05) are shown in bold.

## Data Availability

The data that support the findings of this study are available from the corresponding author upon reasonable request. Processed gait variables and analysis scripts are available for academic use. Due to ethical restrictions, raw participant data containing identifiable information cannot be publicly shared.

## Supplementary Materials

The following supporting information can be downloaded at: https://www.mdpi.com/article/10.3390/s26030820/s1, Supplementary Table S1. Test–retest reliability of gait quality parameters during habitual and fast walking in hip osteoarthritis (OA) and control groups.

## Abbreviations

The following abbreviations are used in this manuscript:

OA	Osteoarthritis
40 m FPWT	40-m fast-paced walk test
IMUs	Inertial measurement units
ACL	Anterior cruciate ligament
SaEn	Sample entropy
MSE	Multiscale entropy
SPARC	Spectral arc length
BMI	Body mass index
VT	Vertical
ML	Mediolateral
AP	Anteroposterior

## References

[1] Woolf A.D., Pfleger B. (2003). Burden of Major Musculoskeletal Conditions. Bull. World Health Organ..

[2] Fu M., Zhou H., Li Y., Jin H., Liu X. (2022). Global, Regional, and National Burdens of Hip Osteoarthritis from 1990 to 2019: Estimates from the 2019 Global Burden of Disease Study. Arthritis Res. Ther..

[3] Murphy L.B., Helmick C.G., Schwartz T.A., Renner J.B., Tudor G., Koch G.G., Dragomir A.D., Kalsbeek W.D., Luta G., Jordan J.M. (2010). One in Four People May Develop Symptomatic Hip Osteoarthritis in His or Her Lifetime. Osteoarthr. Cartil..

[4] Constantinou M., Barrett R., Brown M., Mills P. (2014). Spatial-Temporal Gait Characteristics in Individuals with Hip Osteoarthritis: A Systematic Literature Review and Meta-Analysis. J. Orthop. Sports Phys. Ther..

[5] Bahl J.S., Nelson M.J., Taylor M., Solomon L.B., Arnold J.B., Thewlis D. (2018). Biomechanical Changes and Recovery of Gait Function After Total Hip Arthroplasty for Osteoarthritis: A Systematic Review and Meta-Analysis. Osteoarthr. Cartil..

[6] Porta M., Pau M., Leban B., Deidda M., Sorrentino M., Arippa F., Marongiu G. (2021). Lower Limb Kinematics in Individuals with Hip Osteoarthritis During Gait: A Focus on Adaptative Strategies and Interlimb Symmetry. Bioengineering.

[7] Colyer S.L., Evans M., Cosker D.P., Salo A.I.T. (2018). A Review of the Evolution of Vision-Based Motion Analysis and the Integration of Advanced Computer Vision Methods Towards Developing a Markerless System. Sports Med.-Open.

[8] Dobson F., Hinman R.S., Roos E.M., Abbott J.H., Stratford P., Davis A.M., Buchbinder R., Snyder-Mackler L., Henrotin Y., Thumboo J. (2013). OARSI Recommended Performance-Based Tests to Assess Physical Function in People Diagnosed with Hip or Knee Osteoarthritis. Osteoarthr. Cartil..

[9] Kobsar D., Masood Z., Khan H., Khalil N., Kiwan M.Y., Ridd S., Tobis M. (2020). Wearable Inertial Sensors for Gait Analysis in Adults with Osteoarthritis—A Scoping Review. Sensors.

[10] Benson L.C., Clermont C.A., Bošnjak E., Ferber R. (2018). The Use of Wearable Devices for Walking and Running Gait Analysis Outside of the Lab: A Systematic Review. Gait Posture.

[11] Beck Y., Herman T., Brozgol M., Giladi N., Mirelman A., Hausdorff J.M. (2018). SPARC: A New Approach to Quantifying Gait Smoothness in Patients with Parkinson’s Disease. J. Neuroeng. Rehabil..

[12] Emmerzaal J., Corten K., van der Straaten R., De Baets L., Van Rossom S., Timmermans A., Jonkers I., Vanwanseele B. (2022). Movement Quality Parameters during Gait Assessed by a Single Accelerometer in Subjects with Osteoarthritis and Following Total Joint Arthroplasty. Sensors.

[13] Garcia F.d.V., da Cunha M.J., Schuch C.P., Schifino G.P., Balbinot G., Pagnussat A.S. (2021). Movement Smoothness in Chronic Post-Stroke Individuals Walking in an Outdoor Environment-A Cross-Sectional Study Using IMU Sensors. PLoS ONE.

[14] Paraschiv-Ionescu A., Newman C.J., Carcreff L., Gerber C.N., Armand S., Aminian K. (2019). Locomotion and Cadence Detection Using a Single Trunk-Fixed Accelerometer: Validity for Children with Cerebral Palsy in Daily Life-like Conditions. J. Neuroeng. Rehabil..

[15] Simonetti E., Bergamini E., Bascou J., Vannozzi G., Pillet H. (2021). Three-Dimensional Acceleration of the Body Center of Mass in People with Transfemoral Amputation: Identification of a Minimal Body Segment Network. Gait Posture.

[16] Goshvarpour A., Goshvarpour A. (2012). Nonlinear Analysis of Human Gait Signals. Int. J. Inf. Eng. Electron. Bus..

[17] Moe-Nilssen R., Helbostad J.L. (2004). Estimation of Gait Cycle Characteristics by Trunk Accelerometry. J. Biomech..

[18] Dalton A., Khalil H., Busse M., Rosser A., van Deursen R., Ólaighin G. (2013). Analysis of Gait and Balance Through a Single Triaxial Accelerometer in Presymptomatic and Symptomatic Huntington’s Disease. Gait Posture.

[19] Moraiti C., Stergiou N., Ristanis S., Georgoulis A.D. (2007). ACL Deficiency Affects Stride-to-Stride Variability as Measured Using Nonlinear Methodology. Knee Surg. Sports Traumatol. Arthrosc..

[20] de Oliveira E.A., Andrade A.O., Vieira M.F. (2019). Linear and Nonlinear Measures of Gait Variability After Anterior Cruciate Ligament Reconstruction. J. Electromyogr. Kinesiol..

[21] Pau M., Mandaresu S., Pilloni G., Porta M., Coghe G., Marrosu M.G., Cocco E. (2017). Smoothness of Gait Detects Early Alterations of Walking in Persons With Multiple Sclerosis Without Disability. Gait Posture.

[22] Balasubramanian S., Melendez-Calderon A., Roby-Brami A., Burdet E. (2015). On the Analysis of Movement Smoothness. J. Neuroeng. Rehabil..

[23] van Schooten K.S., Pijnappels M., Rispens S.M., Elders P.J.M., Lips P., Daffertshofer A., Beek P.J., van Dieën J.H. (2016). Daily-Life Gait Quality as Predictor of Falls in Older People: A 1-Year Prospective Cohort Study. PLoS ONE.

[24] Richman J.S., Moorman J.R. (2000). Physiological Time-Series Analysis Using Approximate Entropy and Sample Entropy. Am. J. Physiol. Heart Circ. Physiol..

[25] Sergooris A., Verbrugghe J., Matheve T., Van Den Houte M., Bonnechère B., Corten K., Bogaerts K., Timmermans A. (2023). Clinical Phenotypes and Prognostic Factors in Persons with Hip Osteoarthritis Undergoing Total Hip Arthroplasty: Protocol for a Longitudinal Prospective Cohort Study (HIPPROCLIPS). BMC Musculoskelet. Disord..

[26] Moe-Nilssen R. (1998). A New Method for Evaluating Motor Control in Gait Under Real-Life Environmental Conditions. Part 2: Gait Analysis. Clin. Biomech..

[27] Zijlstra W., Hof A.L. (2003). Assessment of Spatio-Temporal Gait Parameters from Trunk Accelerations During Human Walking. Gait Posture.

[28] Wurdeman S.R. (2016). Lyapunov Exponent. Nonlinear Analysis for Human Movement Variability.

[29] Rosenstein M.T., Collins J.J., De Luca C.J. (1993). A Practical Method for Calculating Largest Lyapunov Exponents from Small Data Sets. Phys. D Nonlinear Phenom..

[30] Mehdizadeh S. (2019). A Robust Method to Estimate the Largest Lyapunov Exponent of Noisy Signals: A Revision to the Rosenstein’s Algorithm. J. Biomech..

[31] Castiglia S.F., Trabassi D., Conte C., Ranavolo A., Coppola G., Sebastianelli G., Abagnale C., Barone F., Bighiani F., De Icco R. (2023). Multiscale Entropy Algorithms to Analyze Complexity and Variability of Trunk Accelerations Time Series in Subjects with Parkinson’s Disease. Sensors.

[32] Farkas G.J., Schlink B.R., Fogg L.F., Foucher K.C., Wimmer M.A., Shakoor N. (2019). Gait Asymmetries in Unilateral Symptomatic Hip Osteoarthritis and Their Association with Radiographic Severity and Pain. Hip Int..

[33] Kiss R.M. (2010). Effect of Walking Speed and Severity of Hip Osteoarthritis on Gait Variability. J. Electromyogr. Kinesiol..

[34] Zhang Y., Li X., Wang Y., Ge L., Pan F., Winzenberg T., Cai G. (2023). Association of Knee and Hip Osteoarthritis with the Risk of Falls and Fractures: A Systematic Review and Meta-Analysis. Arthritis Res. Ther..

[35] Kiko Y., Uchitomi H., Matsubara M., Miyake Y. (2025). Gait Characteristics of Fallers and Nonfallers in Female Patients with Unilateral End-Stage Hip Osteoarthritis. Healthcare.

[36] Yılmaz N., Bağcıer F. (2022). The Evaluation of Postural Stability and Fall Risk in Patients with Primary Hip Osteoarthritis. Indian J. Orthop..

[37] Alkhamis B.A., Reddy R.S., Alahmari K.A., Alshahrani M.S., Koura G.M., Ali O.I., Mukherjee D., Elrefaey B.H. (2024). Balancing Act: Unraveling the Link Between Muscle Strength, Proprioception, and Stability in Unilateral Hip Osteoarthritis. PLoS ONE.

[38] Arnold C.M., Faulkner R.A. (2007). The History of Falls and the Association of the Timed up and Go Test to Falls and Near-Falls in Older Adults with Hip Osteoarthritis. BMC Geriatr..

[39] Mawarikado Y., Uchihashi Y., Inagaki Y., Ogawa M., Uchihara Y., Seriu N., Ishida Y., Kobayashi Y., Sakata A., Nogami K. (2025). Relationship Between History of Falls and Foot Pressure Centre Parameters During Gait and Stance in Patients with Lower-Limb Osteoarthritis. Sci. Rep..

[40] Lin X., Meijer O.G., Lin J., Wu W., Lin X., Liang B., van Dieën J.H., Bruijn S.M. (2015). Frontal Plane Kinematics in Walking with Moderate Hip Osteoarthritis: Stability and Fall Risk. Clin. Biomech..

[41] Yamada M., Hirata S., Ono R., Ando H. (2006). The Assessment of an Abnormal Gait by Gait Parameters Derived from Trunk Acceleration in Patients with Osteoarthritis of the Hip. Phys. Ther. Jpn..

[42] Bhargava P., Shrivastava P., Nagariya S. (2007). Assessment of Changes in Gait Parameters and Vertical Ground Reaction Forces After Total Hip Arthroplasty. Indian J. Orthop..

[43] Ahn S., Choi W., Jeong H., Oh S., Jung T.-D. (2023). One-Step Gait Pattern Analysis of Hip Osteoarthritis Patients Based on Dynamic Time Warping Through Ground Reaction Force. Appl. Sci..

[44] Higgs J.P., Diamond L.E., Saxby D.J., Barrett R.S., Graham D.F. (2023). Individual Muscle Contributions to the Acceleration of the Centre of Mass During Gait in People with Mild-to-Moderate Hip Osteoarthritis. Gait Posture.

[45] Diamond L.E., Hoang H.X., Barrett R.S., Loureiro A., Constantinou M., Lloyd D.G., Pizzolato C. (2020). Individuals with Mild-to-Moderate Hip Osteoarthritis Walk with Lower Hip Joint Contact Forces despite Higher Levels of Muscle Co-Contraction Compared to Healthy Individuals. Osteoarthr. Cartil..

[46] Allison K., Hall M., Wrigley T.V., Pua Y.-H., Metcalf B., Bennell K.L. (2018). Sex-Specific Walking Kinematics and Kinetics in Individuals with Unilateral, Symptomatic Hip Osteoarthritis: A Cross Sectional Study. Gait Posture.

[47] Kang H.G., Dingwell J.B. (2008). Effects of Walking Speed, Strength and Range of Motion on Gait Stability in Healthy Older Adults. J. Biomech..

[48] Wade F., Huang C.-H., Foucher K.C. (2025). Individual Joint Contributions to Forward Propulsion During Treadmill Walking in Women with Hip Osteoarthritis. J. Orthop. Res..

[49] Abidi S.A.R., Anwar K., Ullah Z., Shaikh A.R., Kakar A.U.K., Ahmad S., Siddique A. (2024). Individual Joint Contributions to Forward Propulsion During Treadmill Walking in Women with Hip Osteoarthritis. J. Popul. Ther. Clin. Pharmacol..

[50] Adouni M., Alkhatib F., Hajji R., Faisal T.R. (2024). Effects of Overweight and Obesity on Lower Limb Walking Characteristics from Joint Kinematics to Muscle Activations. Gait Posture.

