# Supplementary material for: Evaluating Gait Quality in People with Hip Osteoarthritis During Habitual and Fast Walking Using a Trunk Inertial Measurement Unit in Clinical Settings
Source: Sensors (Basel). 2026 Jan 26;26(3):820. doi: 10.3390/s26030820 (PMC12899795; doi:10.3390/s26030820)
Supplement: Supplementary file 1 [file sensors-26-00820-s001.zip › sensors-4082710-supplementary.pdf]

Supplementary Table S1. Test–retest reliability of gait quality parameters during habitual and fast walking in hip osteoarthritis (OA) and control groups.

	Habitual walking			Fast walking		
	S1	S2	ICC	S1	S2	ICC
**Step symmetry**						
VT	0.839 ± 0.077	0.833 ± 0.087	0.788	0.755 ± 0.082	0.749 ± 0.096	0.934
ML	0.612 ± 0.117	0.607 ± 0.125	0.968	0.603 ± 0.094	0.599 ± 0.092	0.968
AP	0.780 ± 0.092	0.778 ± 0.090	0.816	0.657 ± 0.101	0.648 ± 0.101	0.949
**Stride symmetry**						
VT	0.812 ± 0.073	0.821 ± 0.065	0.789	0.664 ± 0.120	0.658 ± 0.110	0.792
ML	0.616 ± 0.104	0.620 ± 0.121	0.79	0.531 ± 0.115	0.519 ± 0.091	0.79
AP	0.750 ± 0.087	0.758 ± 0.079	0.808	0.565 ± 0.106	0.555 ± 0.109	0.83
**Stability**						
VT	1.158 ± 0.143	1.119 ± 0.122	0.931	1.422 ± 0.184	1.426 ± 0.158	0.838
ML	1.081 ± 0.166	1.074 ± 0.146	0.895	1.170 ± 0.145	1.165 ± 0.114	0.791
AP	1.165 ± 0.147	1.138 ± 0.148	0.836	1.369 ± 0.125	1.357 ± 0.122	0.816
**Smoothness**						
Yaw	-1.334 ± 0.096	-1.338 ± 0.095	0.79	-1.325 ± 0.093	-1.328 ± 0.083	0.929
Pitch	-1.412 ± 0.114	-1.407 ± 0.120	0.824	-1.298 ± 0.088	-1.290 ± 0.087	0.789
Roll	-1.573 ± 0.195	-1.581 ± 0.187	0.792	-1.436 ± 0.136	-1.437 ± 0.149	0.816
**Regularity**						
VT	0.753 ± 0.131	0.757 ± 0.128	0.79	0.943 ± 0.184	0.947 ± 0.166	0.88
ML	1.084 ± 0.191	1.072 ± 0.167	0.864	1.057 ± 0.144	1.051 ± 0.116	0.792
AP	0.715 ± 0.218	0.717 ± 0.202	0.852	0.781 ± 0.200	0.799 ± 0.194	0.847
**Complexity**						
VT	0.624 ± 0.158	0.631 ± 0.163	0.831	0.808 ± 0.244	0.810 ± 0.221	0.882
ML	1.224 ± 0.217	1.211 ± 0.175	0.811	1.159 ± 0.171	1.147 ± 0.141	0.82
AP	0.878 ± 0.220	0.849 ± 0.177	0.792	0.912 ± 0.143	0.908 ± 0.154	0.788
